# Effects of Different Doses of sUV-B Exposure on Taxane Compounds’ Metabolism in *Taxus wallichiana* var. *Mairei*

**DOI:** 10.3390/ijms25126407

**Published:** 2024-06-10

**Authors:** Weixue Zhong, Xuchen Tian, Ye Zhang, Xiaoqing Tang, Siqiu Xiao, Ying Zhang, Jing Yang, Ying Liu, Dewen Li

**Affiliations:** 1 College of Chemistry, Chemical Engineering and Resource Utilization, Northeast Forestry University, Harbin 150040, China; 2023120977@nefu.edu.cn (W.Z.); 2021121466@nefu.edu.cn (X.T.); zyzy1111@nefu.edu.cn (Y.Z.); 2023120862@nefu.edu.cn (X.T.); q18287386875@nefu.edu.cn (S.X.); zy-@nefu.edu.cn (Y.Z.); 2022120967@nefu.edu.cn (J.Y.); 2 Key Laboratory of Forest Plant Ecology, Ministry of Education, Northeast Forestry University, Harbin 150040, China; 3 Engineering Research Center of Forest Bio-Preparation, Ministry of Education, Northeast Forestry University, Harbin 150040, China

**Keywords:** *Taxus wallichiana* var. *Mairei*, supplemental UV-B exposure, physiological response, specific secondary metabolites

## Abstract

UV-B is an important environmental factor that differentially affects plant growth and secondary metabolites. The effects of supplemental ultraviolet-B (sUV-B) exposure (T1, 1.40 kJ·m^−2^·day^−1^; T2, 2.81 kJ·m^−2^·day^−1^; and T3, 5.62 kJ·m^−2^·day^−1^) on the growth biomass, physiological characteristics, and secondary metabolites were studied. Our results indicated that leaf thickness was significantly (*p* < 0.05) reduced under T3 relative to the control (natural light exposure, CK); The contents of 6-BA and IAA were significantly reduced (*p* < 0.05); and the contents of ABA, 10-deacetylbaccatin III, and baccatin III were significantly (*p* < 0.05) increased under T1 and T2. The paclitaxel content was the highest (0.036 ± 0.0018 mg·g^−1^) under T3. The cephalomannine content was significantly increased under T1. *Hmgr* gene expression was upregulated under T1 and T3. The gene expressions of *Bapt* and *Dbtnbt* were significantly (*p* < 0.05) upregulated under sUV-B exposure, and the gene expressions of *CoA*, *Ts*, and *Dbat* were significantly (*p* < 0.05) downregulated. A correlation analysis showed that the 6-BA content had a significantly (*p* < 0.05) positive correlation with *Dbat* gene expression. The IAA content had a significantly (*p* < 0.05) positive correlation with the gene expression of *Hmgr*, *CoA*, *Ts*, and *Dbtnbt*. The ABA content had a significantly (*p* < 0.05) positive correlation with *Bapt* gene expression. *Dbat* gene expression had a significantly (*p* < 0.05) positive correlation with the 10-deacetylbaccatin content. *Hmgr* gene expression was positively correlated with the contents of baccatin III and cephalomannine. *Bapt* gene expression had a significantly (*p* < 0.01) positive correlation with the paclitaxel content. A factor analysis showed that the accumulation of paclitaxel content was promoted under T2, which was helpful in clarifying the accumulation of taxane compounds after sUV-B exposure.

## 1. Introduction

*Taxus wallichiana* var. *Mairei*, a member of the *Taxaceae*, is a subtropical coniferous evergreen tree mainly endemic to southern China [1]. It is a unique natural anticancer plant in China and is one of the national-level protected plants [2]. Paclitaxel, a natural diterpenoid extracted from *Taxaceae*, is an important natural anticancer, antitumor, or cytotoxic chemotherapeutic agent with promising efficacy against a wide range of cancers whose manufacturing relies mainly on its extraction from *Taxus wallichiana* var. *Mairei* [3,4]. In recent years, *Taxus wallichiana* var. *Mairei* has been scarce and slow-growing; plant-derived paclitaxel suffers from a short supply due to its low abundance in *Taxus*, limiting its clinical application [5,6], and it is difficult to satisfy the needs of patients for purely natural paclitaxel sources; therefore, in order to solve the supply problem of paclitaxel, it is of great significance to cultivate the growth of *Taxaceae* plants and to increase the research on the response of *Taxaceae* to sUV-B exposure, which is important for the conservation and restoration of *Taxaceae* germplasm resources. 

Ultraviolet-B (UV-B, 280–315 nm) is an important component of the solar exposure spectrum and affects plant survival and adaptation, with the functions of disinfecting as well as sterilizing, and inhibiting plant overgrowth [7]. Stratospheric ozone depletion increases the amount of solar UV-B exposure reaching the Earth’s surface, significantly affecting the secondary metabolism of plants [8]. UV-B radiation can be used as a stress; most studies on UV-B light have focused on the adverse effects of excessive UV-B radiation on plants, and a few have focused on the advantage of supplemental UV-B radiation. Experimental studies have shown that proper UV-B radiation can modulate stem extension and phototropism, enhance leaf area, and promote plant growth [9]. Low-dose sUV-B exposure can improve the contents of plant secondary metabolites, trigger the expression of plant secondary metabolite genes, and elicit morphogenesis as well as defense in plants [10,11]. High-dose sUV-B exposure will cause dwarf plants, shorten the branch pitch, and inhibit flowering [12]. Different doses of sUV-B exposure have different effects on plants.

*Taxaceae* contains hundreds of paclitaxane diterpenoids [13]. 10-Deacetylbaccatin III and baccatin III are paclitaxel diterpenoids isolated and extracted from the leaves of the short branches of *Taxus wallichiana* var. *Mairei*, and are intermediate products for the synthesis of paclitaxel in addition to important raw materials for the synthesis of paclitaxel via the semisynthetic method. They play an irreplaceable role in alleviating the shortage of paclitaxel resources [14]. Cephalomannine is a paclitaxel homologue of paclitaxel, with identical parent nuclei and only slight differences in the side chain portion, which can be structurally modified to transform into paclitaxelcephalomannine, which is structurally very similar to paclitaxel and also possesses strong antitumor activity [15]. Furthermore, in order to solve the resource shortage of *Taxus wallichiana* var. *Mairei*, an understanding of the biosynthesis of *Taxaceae* was essential for exploring other alternative methods of production [16]. Obtaining paclitaxel and its derivatives via semisynthesis using paclitaxel analogues from *Taxaceae* as precursors was one of the most effective methods available [17]. Some studies have shown that branches and leaves were the key parts of renewable *Taxaceae* resources for the extraction of paclitaxel, and while people extract and separate the *Taxus wallichiana* var. *mairei* branches and leaves to obtain paclitaxel. They will also obtain by products, mainly tritoninine, in larger yields than that of paclitaxel, and if they were converted into paclitaxel and its derivatives this would greatly increase the utilization rate of *Taxaceae* resources and reduce the cost of production [18]. Therefore, the four secondary metabolites were important in terms of investigating the effects of sUV-B exposure on them.

We investigated doses of sUV-B exposure as the main influencing factor, examined their effect on the change in the content of paclitaxel-like constituents of *Taxus wallichiana* var. *Mairei*, and screened out the optimal dose of exposure in order to provide a basis for the determination of the optimal growth environment of *Taxus wallichiana* var. *Mairei*. 

## 2. Results

### 2.1. Effects of Different Doses of sUV-B Exposure on the Growth Biomass and Leaf Characteristics of Taxus wallichiana var. Mairei

**Figure 1:**
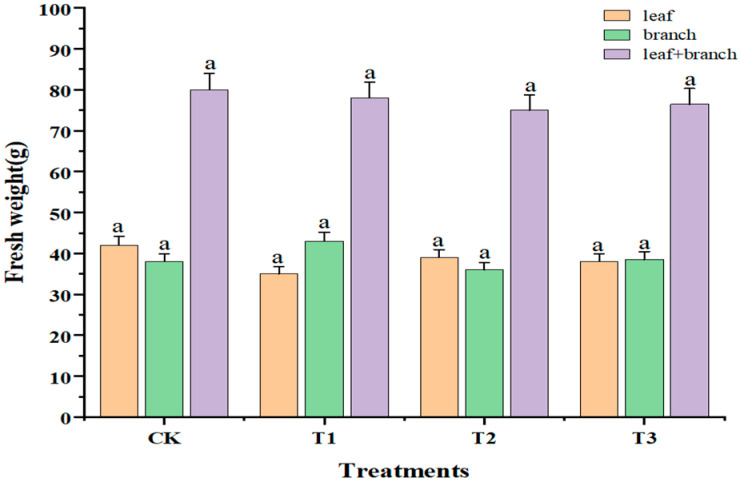
Changes in the fresh weight of leaves and branches under sUV-B exposure. Different lowercase letters in the same column represent a significant difference (*p* < 0.05), and the same lowercase letters in the same column represent no significant difference (*p* > 0.05).

With increasing doses of sUV-B exposure, the color of leaves gradually turned yellow with the phenomenon of leaf drop. Compared with CK, the leaves showed obvious chlorosis under the T3 treatment (5.62 kJ·m^−2^·day^−1^), which affected the growth of the plant. The effect of sUV-B exposure on the fresh weight of *Taxus wallichiana* var. *Mairei* is shown in Figure 1. The fresh weight and total fresh weight of leaves and branches decreased after sUV-B exposure, but there was no significant difference between the treatments (*p* < 0.05).

As shown in Table 1, compared with CK, plant height became slightly shorter; the leaf length, leaf width, leaf area, leaf thickness, and leaf water content of *Taxus wallichiana* var. *Mairei* decreased after sUV-B exposure; and leaf thickness and leaf water content were significantly different (*p* < 0.05) under the T3 treatment. 

In summary, our results are showed that the plant growth and leaf characteristics were not obviously affected in response to short-term and low-dose sUV-B exposure.

### 2.2. Effect of Different Doses of sUV-B Exposure on the Endogenous Hormone Content of Taxus wallichiana var. Mairei

**Figure 2:**
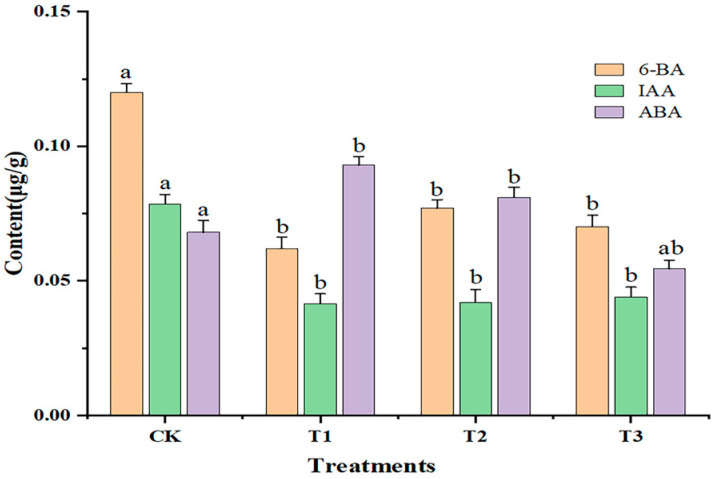
Changes in the endogenous hormone content of leaves under sUV-B exposure. Different lowercase letters in the same column represent a significant difference (*p* < 0.05), and the same lowercase letters in the same column represent no significant difference (*p* > 0.05).

As shown in Figure 2, compared with CK, the contents of 6-BA and IAA were significantly decreased (*p* < 0.05) with increasing doses of sUV-B exposure. The content of 6-BA was the lowest (0.062 ± 0.011 μg·g^−1^) under the T1 treatment. The IAA content under T1, T2, and T3 was lower than that under CK. The ABA content was significantly increased (*p* < 0.05) under T1 and T2, while the ABA content was significantly decreased (*p* < 0.05) under T3. Thus, the contents of both 6-BA and IAA were inhibited under sUV-B exposure.

### 2.3. Effect of Different Doses of sUV-B Exposure on the Taxane Compound Contents of Taxus wallichiana var. Mairei

**Figure 3:**
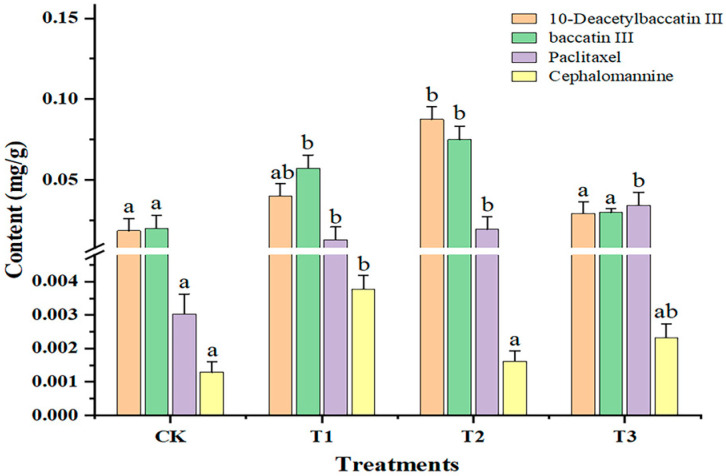
Changes in the secondary metabolite content of leaves under sUV-B exposure. Different lowercase letters in the same column represent a significant difference (*p* < 0.05), and the same lowercase letters in the same column represent no significant difference (*p* > 0.05).

As shown in Figure 3, compared with CK, the 10-deacetylbaccatin III content was significantly increased (*p* < 0.05) under T2 and the 10-deacetylbaccatin III content was highest under T2 (0.089 μg·g^−1^ ± 0.0078 mg·g^−1^). The baccatin III content was significantly increased (*p* < 0.05) under T1 and T2, and the changing trend in baccatin III content was T2 > T1 > T3 > CK (T1, 1.40 kJ·m^−2^·day^−1^; T2, 2.81 kJ·m^−2^·day^−1^; and T3, 5.62 kJ·m^−2^·day^−1^). With increasing doses of sUV-B exposure, the paclitaxel content was significantly increased (*p* < 0.05), of which the paclitaxel content was highest (0.036 μg·g^−1^ ± 0.0018 mg·g^−1^) under T3. The content of cephalomannine increased 3.8-fold, 1.29-fold, and 2.3-fold under T1, T2, and T3, respectively. Based on our results, we propose that the low-dose sUV-B exposure (1.40 kJ·m^−2^·d^−1^) promoted the cephalomannine content. Furthermore, the three sUV-B exposures significantly promoted the paclitaxel content (*p* < 0.05).

### 2.4. Effect of Different Doses of sUV-B Exposure on the Expression of Key Genes for Paclitaxel Synthesis

**Figure 4:**
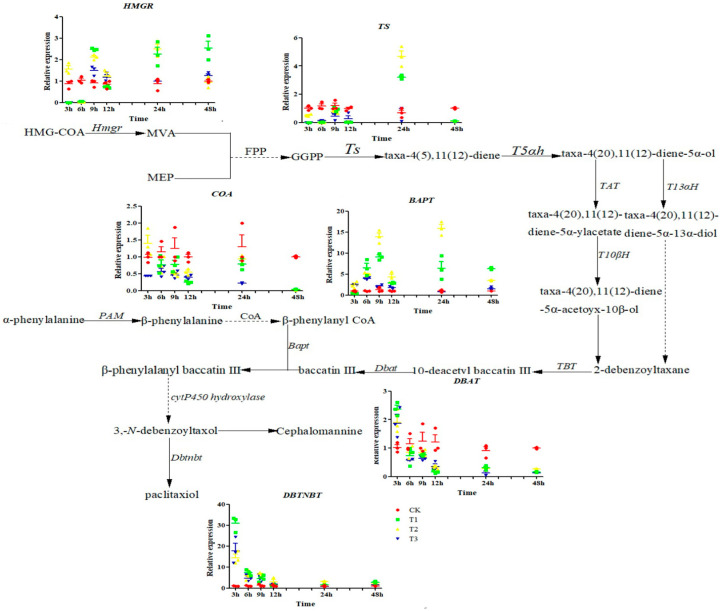
Changes in the gene expression of the biosynthetic pathway under sUV-B exposure. Reprinted with permission from Ref. [19]. 2016, Kuang, X.J.; Wang, C.X.; Zou, L.Q.; Li, Y.; Sun, C.

As shown in Figure 4, with increasing doses of sUV-B exposure, *hmgr* gene expression was upregulated under T1 and T3, while gene expression was significantly down-regulated (*p* < 0.05) under T2. Compared with CK, *CoA* gene expression was significantly (*p* < 0.05) downregulated under T1, T2, and T3; however, the change in *CoA* gene expression under T2 was significantly upregulated (*p* < 0.05), with an upregulation of 29.8%. The gene expressions of *Ts* and *Dbat* under different sUV-B exposures saw downregulation trends. While the gene expressions of *Bapt* and *Dbtnbt* were significantly upregulated under T1, T2, and T3 (*p* < 0.05), with the most significant upregulation trend in gene expression being under T1.

### 2.5. Correlation Analysis between Endogenous Hormone Content, Secondary Metabolite Content, and the Expression of Key Enzyme Genes for Paclitaxel Synthesis

**Figure 5:**
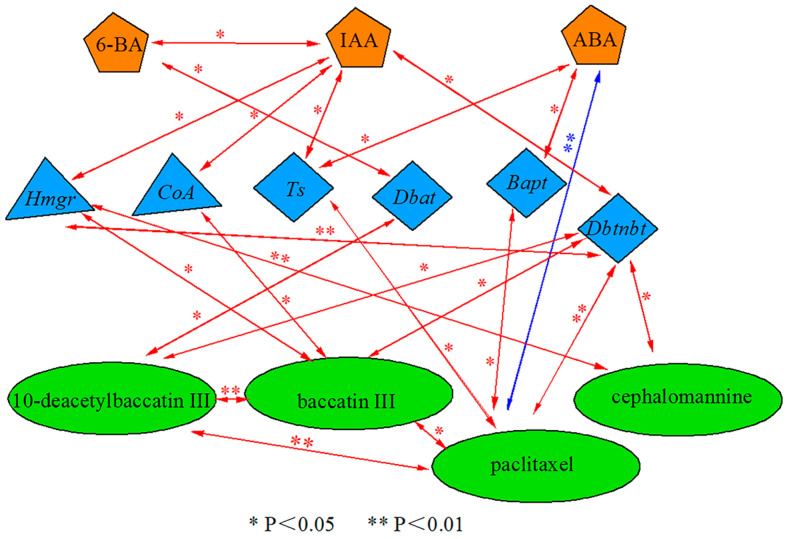
Correlation analysis of the endogenous hormone content, taxane compound content, and the expression of key genes for paclitaxel synthesis in *Taxus wallichiana* var. *Mairei*. (red arrows indicate a strong positive correlation; blue arrows indicate a strong negative correlation).

Significant differences were found between endogenous hormone contents, secondary metabolite contents of *Taxus wallichiana* var. *Mairei*, and the expression of key enzyme genes for paclitaxel synthesis after sUV-B exposure (Figure 5). The content of 6-BA was significantly (*p* < 0.05) positively correlated with *Dbat* gene expression, and *Dbat* gene expression was significantly (*p* < 0.05) positive correlated with the 10-deacetylbaccatin content. The IAA content was significantly (*p* < 0.05) positive correlated with upstream synthesis pathway genes (*Hmgr*, *CoA*), which positively correlated with the baccatin III content. *Hmgr* gene expression was also significantly (*p* < 0.01) positive correlated with the cephalomannine content. Furthermore, the IAA content was significantly (*p* < 0.05) positive correlated with downstream synthesis pathway genes (*Ts*, *Dbtnbt*), which were significantly (*p* < 0.01) positive correlated with the paclitaxel content. The ABA content was significantly (*p* < 0.05) positive correlated with *Bapt* gene expression, and *Bapt* gene expression was significantly (*p* < 0.05) positive correlated with the paclitaxel content. The ABA content was significantly (*p* < 0.01) negative correlated with the paclitaxel content.

In conclusion, our study mainly investigated changes in the endogenous hormone 6-BA content regulating downstream pathway synthesis (*Dbat*) gene expression, which in turn affects the 10-deacetylbaccatin content, and changes in the endogenous hormone IAA content regulating upstream pathway synthesis gene (*Hmgr*, *CoA*) expression, which in turn affects the baccatin III content. The IAA content also regulates downstream pathway synthesis gene (*Ts*, *Dbtnbt*) expression, which in turn affects the contents of paclitaxel and cephalomannine.

### 2.6. Comprehensive Evaluation Based on Factor Analysis 

The factor analysis extracted three ingredients, and the ingredient score coefficient matrix was obtained as shown in Table 2. The first common factor mainly represented the plant height, leaf width, leaf area, IAA content, baccatin III content, cephalomannine content, *CoA* gene expression, *Ts* gene expression, *Hmgr* gene expression, and *Dbtnbt* gene expression. The second common factor mainly represented the leaf length, ABA content, paclitaxel content, and *Bapt* gene expression. The third common factor mainly represented the leaf water content, 6-BA content, 10-deacetylbaccatin III content, and *Dbat* gene expression.

According to the factor score model, composite scores under different sUV-B exposures were obtained. As shown in Table 3, in the first ingredient score, the score of T1 was significantly higher than that of the other treatments; in the second ingredient score, the score of T2 was significantly higher than that of the other treatments; and, in the third ingredient score, the score of CK was significantly higher than that of the other treatments. The composite scores indicated that T2 had a better composite score than the other treatments. The composite scores of different doses of sUV-B exposure were as follows: T2 > T1 > CK > T3.

## 3. Discussion

The measurement of physiological indexes has great significance in studying the growth of *Taxus wallichiana* var. *Mairei* and guiding production under sUV-B exposure, it is a common index in plant research. Although plants are inevitably exposed to UV-B light, they are able to modulate their light response to optimize growth [20]. Morphological changes, such as reducing leaf area, increasing leaf thickness, and shortening internodes as well as petiole lengths were frequently observed in plants exposed to UV-B exposure [21]. The study by Wang Dan et al. [22] found that under natural light + UV-B, Z. pendula leaf area decreased but leaf thickness increased. This is the same as our findings that *Taxus wallichiana* var. *Mairei* leaves resist UV-B exposure by reducing their area under sUV-B exposure. UV-B light can be used as an information signal with which to regulate plant development and as a potential environmental threat to plant survival UV-B [23]. Several studies have found that growth parameters, such as the plant height and leaf area of wheat, rice, maize, rye, soybean, sunflower, cucumber, and spinach were significantly decreased under high-dose UV-B exposure [24]. The results showed that the changes in the plant height, leaf weight, and branch weight of *Taxus wallichiana* var. *Mairei* all showed a decreasing trend with increasing doses of sUV-B exposure. This is consistent with our experimental results that low-dose sUV-B exposure (1.40 kJ·m^−2^·day^−1^) promoted the growth and metabolism of *Taxus wallichiana* var. *Mairei*. Chu Run et al. [25] found that, under short-term UV-B exposure (1.68 kJ·m^−2^·day^−1^), the height and biomass of *Typha orientalis* increased significantly. A study has found that tomato plant height decreased, and that stem diameter, crown, and root dry mass as well as the seedling index first increased and then decreased with increasing doses of UV-B exposure (1.45 kJ·m^−2^·day^−1^–5.68 kJ·m^−2^·day^−1^) and time [26]. The results of Qi Wencai et al. [27] showed that short-term and low-dose sUV-B exposure had no significant effect on the plant growth and yield of purple potato tubers. In this experiment, the growth and leaf characteristics of *Taxus wallichiana* var. *Mairei* under T1 and T2 were not changed compared with CK, while the decreasing trend under T3 was obvious, indicating that high-dose sUV-B exposure was unfavorable to the growth of *Taxus wallichiana* var. *Mairei*. This was the same as the findings of Zhu Xudong et al. [28]. sUV-B exposure could alter the morphological structure of the plant and induce a large number of defense secondary metabolites to mitigate sUV-B exposure or enhance resistance. The metabolic pathways in plants were intricate and complex, and there were many cross-linkages between the pathways, so the formation of secondary metabolites in the pathway also produced a variety of hormone synthesis prerequisites [29], such as 6-BA, IAA, and ABA, synthesized by the isoprenoid pathway. Some studies have shown that [30] different sUV-B exposures significantly reduced the dry height of *Taxus wallichiana* var. *Mairei*, shortened the length of branches, reduced the number of leaves, and lowered the levels of tryptophan (TRP) as well as IAA. ABA was closely linked to the regulation of secondary metabolite synthesis and accumulation, which can be regulated by a variety of factors, including synthetic pathways, hormonal interactions, and signals [31]. Our experiment showed that the contents of 6-BA and IAA were decreased under different sUV-B exposures. In this study, we also found that the ABA content was significantly increased under T1 (1.40 kJ·m^−2^·day^−1^). Related studies have shown that the ABA content was increased under different sUV-B exposures in tomato seedlings [32]. The study showed that the changes in secondary metabolite content in *Taxus wallichiana* var. *Mairei* were closely related to the adversity stress to which a plant was subjected [33]. Our results showed that fresh leaves of *Taxus wallichiana* var. *Mairei* were subjected to adverse stress under sUV-B exposure, which led to an increase in the contents of paclitaxel and cephalomannine. This may be due to the fact that sUV-B exposure regulates the enzyme activities of pathway genes in the metabolic process, and thus the *Taxus wallichiana* var. *Mairei* leaves accumulate more paclitaxel in response to the stress, which was in agreement with the study of Yang Fengjian et al. [34]. In contrast, the paclitaxel content increased in the leaves of *Taxus wallichiana* var. *Mairei* with the prolongation of sUV-B exposure. This may be related to 10-deacetylbaccatin III and baccatin III. As a diterpenoid, paclitaxel was obtained through a series of enzymatic reactions during photosynthesis in *Taxus wallichiana* var. *Mairei*, and its metabolism was regulated by many metabolic enzymes. It has been reported [35] that increasing the expression level of the *Ts* gene facilitates the promotion of paclitaxel accumulation. *Ts* was thought to be a key enzyme gene that catalyzes paclitaxel synthesis [36]. The relationship between the expression of the *Dbat*, *Bapt,* and *Dbtnbt* genes was thought to be involved in the early and late steps of the paclitaxel pathway [37]. Our results showed that the expression of the *Hmgr* gene was significantly increased under different sUV-B exposures, and its relative expression was generally two–three-fold higher than that of CK, which plays a decisive role in the synthesis of MVA. The *Dbtnbt* gene was an enzyme gene required for catalyzing the last step of paclitaxel biosynthesis and is responsible for catalyzing the formation of paclitaxel from the paclitaxel precursor with an incomplete side chain, which determines to a certain extent the amount of synthesis of the end product paclitaxel [38,39,40]. There was a negative correlation between the growth and the paclitaxel content, but the correlation was not significant, which indicated that paclitaxel may be a defense substance that makes *Taxus wallichiana* var. *Mairei* adapt to adversity, such as high temperature, drought, high light intensity, and so on.

Correlation analysis is a statistical analysis tool that was used to analyze the correlation between two sets of random variables [41]. The factor analysis took the correlation of the original variables as the starting point, and used the idea of dimensional reduction to simplify the multivariate variables into a small number of factors by exploring the internal relationships of the original variable matrix and then analyzing their intrinsic associations [42]. In summary, the results of the factor analysis were consistent with the results of the correlation analysis (Figure 5, Table 3). With increasing doses of sUV-B exposure, the *Hmgr*, *Ts*, and *Bapt* genes were highly expressed under the T2 treatment. The content of the secondary metabolite paclitaxel was the highest at 24 h. This suggests that sUV-B exposure (T2, 2.81 kJ·m^−2^·day^−1^) regulated the high expression of paclitaxel pathway genes and improved the production of secondary metabolites, thereby favoring the production of *Taxus wallichiana* var. *Mairei*.

## 4. Materials and Methods

### 4.1. Plant Materials and sUV-B Exposure

**Figure 6:**
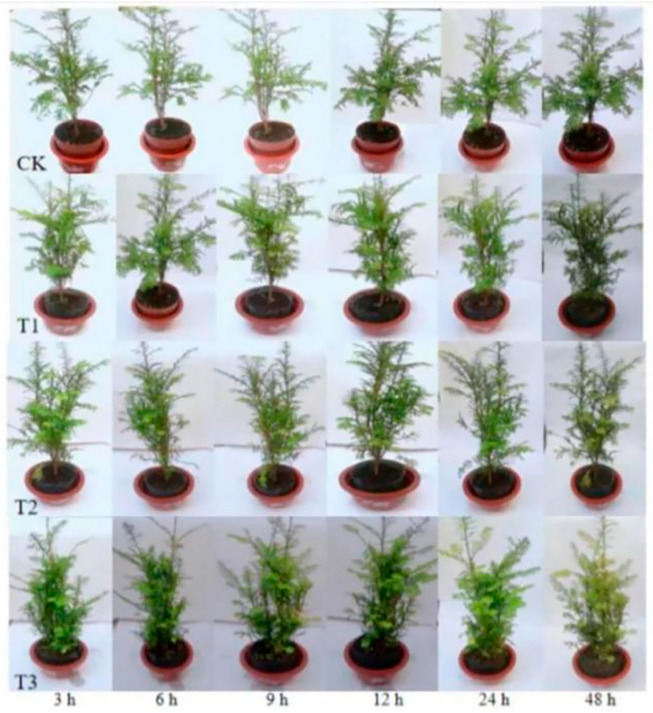
sUV-B exposure on the morphological characterization of *Taxus wallichiana* var. *Mairei*.

This experiment was conducted outdoors at the Northeast Forestry University, Heilongjiang Province, China (45.75° N, 126.63° E, and 1826 m above sea level), for the *Taxus wallichiana* var. *Mairei* seedlings (growing season was from 21 April 2018 to 21 November 2018). Four groups of experiments were designed, with 10 pots (11 × 8 × 15 cm) in each group. The basic physical and chemical properties of the tested soil were as follows: pH of 5.57; organic matter, 184.85 g kg^−1^; alkali-hydrolyzable nitrogen, 438.9 mg kg^−1^; and available phosphorus, 11.05 mg kg^−1^. The first group was the control group (CK), which grew in natural light. In the treatments, under the same growth conditions, the UV-B (280–320) exposure treatment was generated by a 40 W fluorescent lamp (purchased from the Beijing Research Institute of Electric Light Source (Beijing, China)) hanging above the plant. The doses of UV-B radiation was measured via AvaSpec 2048-2 (Avantes BV, Beijing, China); they were 1.40 kJ·m^−2^·day^−1^ (T1), 2.81 kJ·m^−2^·day^−1^ (T2), and 5.62 kJ·m^−2^·day^−1^ (T3), respectively. The emitted ultraviolet rays are filtered by a 0.08 mm cellulose acetate membrane, in order to eliminate the interference caused by the influence of UV-C. The plants under sUV-B exposure were treated for 3 h, 6 h, 9 h, 12 h, 24 h, and 48 h (Figure 6); we selected the plants treated for 48 h as materials. Daily management was carried out during the test period to ensure the supply of water and nutrients.

### 4.2. Measurement of Growth Biomass and Physiological Characteristics of Taxus wallichiana var. Mairei

Five *Taxus wallichiana* var. *Mairei* were randomly selected in the experimental treatment group and a straightedge was used to determine plant height, leaf length, and leaf width of *Taxus wallichiana* var. *Mairei* seedlings. Leaf area was analyzed with a portable photosynthetic system (LI-6400 model, California, USA). Leaf thickness was measured using a semithin cross-section cut from the leaf material using a micrometer (7331MEXRL-25 model, Jiangsu, China). Leaf water content was calculated by fresh and dry weight: leaf water content = (fresh weight − dry weight)/fresh weight.

### 4.3. Measurement of the Endogenous Hormone Content of Taxus wallichiana var. Mairei

The contents of 6-BA, IAA, and ABA were determined using high-performance liquid chromatography (HPLC, Agilent 1260 liquid chromatography system, California, USA). Sample processing: 2 g of fresh samples of *Taxus wallichiana* var. *Mairei* were placed in a mortar, ground with liquid nitrogen, transferred into a 15 mL centrifuge tube, and pre-cooled with 80% methanol 4 °C. After being added to 15 mL of 80% methanol pre-cooled at 4°C, ultrasonic extraction was carried out for 10 h. After centrifuging the samples at 5000 rpm for 15 min, the suspension was taken, and the residue was extracted twice with the above method, and then combined with the suspension. The suspension was evaporated and concentrated to 2–3 mL at 35 °C; the concentrate was aspirated, washed with chromatographically pure methanol in small quantities for several times, fixed to 10 mL with methanol, passed through a 0.22 mL filter membrane, and left to be measured. The chromatographic conditions were as follows: C18 (150 mm × 46 mm, 5 Mm) (Diamonsil, Beijing, China). The mobile-phase conditions were as follows: methanol-0.6% acetic acid (50:50). The column temperature was 35 °C; the detection wavelength was 254 nm; and the flow rate was 1 mL·min^−1^.

### 4.4. Measurement of the Specific Secondary Metabolite Contents of Taxus wallichiana var. Mairei

The contents of 10-deacetylbaccatin III, baccatin III, paclitaxel, and cephalomannine were determined using high-performance liquid chromatography (HPLC, Agilent 1260 liquid chromatography system, USA) according to the method of Loganathan D et al. [43]. Sample processing was carried out as follows: 0.5 g of fresh sample was added to 10 mL of methanol and underwent ultrasonic extraction for 20 min, and was then centrifuged for 10 min (8000 rm·min^−1^); the suspension was transferred to a 15 mL centrifuge tube, and the residue was added to 10 mL of methanol, shaken well, and then ultrasonic extraction was carried out for 20 min and centrifuged for 10 min (8000 rm·min^−1^) and then combined with the suspension, which was then dried (about 4 h) in negative-pressure concentration and volatile machine. The suspension was then dried in a negative-pressure concentrator (about 4 h). After evaporation, 1 mL of methanol was added to the centrifuge tube to reconstitute the solution (sonicated for 1 h); the reconstituted solution was transferred into a 1.5 mL centrifuge tube and centrifuged for 10 min (12,000 rm·min^−1^), and the suspension was taken for measurement. Finally, 10 μL of each sample was injected into the HPLC. Chromatographic conditions: C18 (5 μm, 4.6 mm × 250 mm) (Diamonsil, China); mobile-phase conditions: acetonitrile/water = 37/63 (*v*:*v*); column temperature: 35 °C; detection wavelength: 234 nm; and flow rate: 1 mL·min^−1^.

### 4.5. Measurement of Gene Expression of Key Enzymes in Paclitaxel Synthesis of Taxus wallichiana var. Mairei

Total RNA was extracted by a modified CTAB-LiCl coupling method [44], and cDNA was synthesized using a BeyoRT™ II cDNA first strand synthesis kit (Beyotime Biologicals, Nanjing, China). Using the NCBI database, the sequences of the genes related to the paclitaxel synthesis pathway were found, and it was applied to design the primers, and the primer sequences were shown in Table 4. The cDNA was used as the template, and the gene 18S rRNA was used as the primer for PCR amplification [45]. Of the total PCR reaction system, 20 μL was used: 2 μL of template cDNA, 1.5 μL of each of the primer 18S, 10 μL of PCR-Mix (Easy-Load™ PCR Master Mix, Biyuntian Biologicals, Nanjing, China), and 5 μL of ddH_2_O. PCR amplification system: 94 °C for 3 min, 94 °C for 30 s, 55 °C for 30 s, 72 °C for 1 min, 30 cycles, 72 °C for 10 min, and keep warm at 4 °C. PCR amplification products were detected by 1.2% agarose gel electrophoresis. The relative expression of target genes was calculated by Delta-deta Ct.

### 4.6. Factor Analysis

Nineteen indicators of influencing factors were selected, namely plant height (Q1), leaf length (Q2), leaf width (Q3), leaf area (Q4), leaf thickness (Q5), leaf water content (Q6), 6-BA content (Q7), IAA content (Q8), ABA content (Q9), 10-deacetylbaccatin III content (Q10), bacitracin III content (Q11), paclitaxel content (Q12), cephalomannine content (Q13), *CoA* gene expression (Q14), *Ts* gene expression (Q15), *Hmgr* gene expression (Q16), *Dbat* gene expression (Q17), *Bapt* gene expression (Q18), and *Dbtnbt* gene expression (Q19), which were subjected to factor analysis.

### 4.7. Data Analysis

The experimental data were analyzed and processed using SPSS 26.0 software, Origin 2022, and Microsoft Excel 2010.

## 5. Conclusions

The results of this study indicated that sUV-B exposure affected the growth and specific secondary metabolism of *Taxus wallichiana* var. *Mairei*. The three sUV-B exposures significantly promoted the paclitaxel content (*p* < 0.05). The paclitaxel content was highest (0.036 μg·g^−1^ ± 0.0018 mg·g^−1^) under T3. The cephalomannine content was increased 3.8-fold, 1.29-fold, and 2.3-fold under T1, T2, and T3, respectively. The IAA content regulated the upstream pathway synthesis gene (*Hmgr*), and the *Hmgr* gene regulated the cephalomannine content in turn. Furthermore, the IAA content regulated the downstream synthesis pathway genes (*Ts*, *Dbtnbt*, and *Bapt*), and the *Bapt* gene regulated the paclitaxel content in turn. This may be due to the gradual decrease in the ABA content. The lower the ABA content, the higher the paclitaxel content. Based on our results, we propose that low-dose sUV-B exposure (1.40 kJ·m^−2^·day^−1^) promoted the *Taxanes* compound content. All in all, our study provides theoretical guidance for promoting the growth and development of *Taxus wallichiana* var. *Mairei*, producing medicinal active components and improving the utilization rate of *Taxus wallichiana* var. *Mairei*. 

## Figures and Tables

**Table 1:** Changes in the physiological characteristics of leaves under sUV-B exposure.

Treatment	Plant Height (cm)	Leaf Length (cm)	Leaf Width (cm)	Leaf Area (cm^2^)	Leaf Thickness (mm)	Leaf Water Content (%)
CK	49.93 ± 1.703 ^a^	9.7 ± 1.422 ^a^	5.4 ± 0.31 ^a^	25.77 ± 2.51 ^a^	0.11 ± 0.0057 ^a^	71.76 ± 0.741 ^ab^
T1	49.73 ± 1.43 ^a^	9.167 ± 0.89 ^a^	5.47 ± 0.145 ^a^	25.12 ± 2.8 ^a^	0.12 ± 0.0033 ^a^	70.39 ± 0.18 ^b^
T2	48.87 ± 0.25 ^a^	9.13 ± 0.98 ^a^	5.43 ± 0.425 ^a^	25.17 ± 4.46 ^a^	0.11 ± 0.001 ^a^	72.85 ± 0.283 ^a^
T3	48.03 ± 1.84 ^a^	9.17 ± 0.84 ^a^	4.63 ± 0.08 ^a^	21.21 ± 1.78 ^a^	0.09 ± 0.001 ^b^	69.02 ± 0.38 ^b^

Note: Values are mean SEM, and the significance of differences between sUV-B treatments was marked with different letters (*p* < 0.05).

**Table 2:** Ingredient score coefficient matrix.

Factor	Ingredient		
	1	2	3
Q1	0.089	0.083	−0.002
Q2	−0.016	0.180	0.025
Q3	0.100	−0.008	0.072
Q4	0.086	0.027	0.083
Q5	0.118	−0.019	0.012
Q6	0.034	−0.019	0.176
Q7	−0.021	−0.174	0.057
Q8	0.121	−0.086	0.002
Q9	−0.023	0.178	0.036
Q10	0.010	0.012	0.185
Q11	0.064	−0.173	0.076
Q12	−0.115	0.116	0.004
Q13	0.075	−0.030	−0.178
Q14	0.092	0.026	−0.157
Q15	0.116	0.009	−0.008
Q16	0.088	0.066	−0.139
Q17	0.018	−0.004	0.184
Q18	0.004	0.170	0.030
Q19	0.113	0.031	−0.039

Note: plant height (Q1), leaf length (Q2), leaf width (Q3), leaf area (Q4), leaf thickness (Q5), leaf water content (Q6), 6-BA content (Q7), IAA content (Q8), ABA content (Q9), 10-deacetylbaccatin III content (Q10), bacitracin III content (Q11), paclitaxel content (Q12), cephalomannine content (Q13), CoA gene expression (Q14), Ts gene expression (Q15), Hmgr gene expression (Q16), Dbat gene expression (Q17), Bapt gene expression (Q18), and Dbtnbt gene expression (Q19).

**Table 3:** Comprehensive scores and ranking of different ingredient indicators.

UV-B Treatment	F1	F2	F3	F	Rank
CK	0.04253	−0.87442	1.21803	0.57	3
T1	1.14458	−0.34742	−0.90512	0.17	2
T2	0.10771	1.43804	0.41285	0.1	1
T3	−1.29481	−0.21621	−0.72576	−0.83	4

F1, F2, F3, and F are the first, second, third, and composite ingredients, respectively.

**Table 4:** Sequences of the primers used to amplify the genes. Primer sequence reference M. Onrubia et al. [46].

Primer	Primer Sequence	PCR Amplicon Size (bp)	Gene ID
*18S*-F	5′-GTGCACATCCCGACTCT-3′	102	AF259290.1
*18S*-R	5′-GCGATCCGTCGAGTTATCAT-3′		
*CoA-ligase*-F	5′-AGCAGACACTATGGAACA-3′	109	KM593667.1
*CoA-ligase*-R	5′-GCCACAACTCTCCTCTAT-3′		
*Hmgr*-F	5′-TAGGGCTCCCGTTGTTAGGT-3′	207	AY544991.1
*Hmgr*-R	5′-TTCATCCCCATGGCATCTCC-3′		
*Ts*-F	5′-TTCGCACGCACGGATACG-3′	115	AY007207.1
*Ts*-R	5′-TTCACCACGCTTCTCAATTCG-3′		
*Dbat*-F	5′-AGTTGGATTTGGTGATCGACTT-3′	92	AY365031.2
*Dbat*-R	5′-ATCCATGTTGCACGAGACTT-3′		
*Bapt*-F	5′-TAAGCACTCTACAACAACAATGG-3′	111	NC003071.7
*Bapt*-R	5′-GCATGAACATTAGTATCTTGATTCC-3′		
*Dbtnbt*-F	5′-CGGGGGGTTTGTTGTGGGATTA-3′	104	AY326950.1
*Dbtnbt*-R	5′-TTAGCCTCTCCCCTCGCCATCT-3′		

## Data Availability

All data are contained within the article.
